# The genome sequence of the Small Ranunculus, *Hecatera dysodea* (Denis & Schiffermüller, 1775)

**DOI:** 10.12688/wellcomeopenres.19067.1

**Published:** 2023-02-23

**Authors:** Gavin R. Broad, Natural History Museum Genome Acquisition Lab, Darwin Tree of Life Barcoding collective, Wellcome Sanger Institute Tree of Life programme, Wellcome Sanger Institute Scientific Operations: DNA Pipelines collective, Tree of Life Core Informatics collective, Darwin Tree of Life Consortium

**Affiliations:** 1 Department of Life Science, Natural History Museum, London, UK

**Keywords:** Hecatera dysodea, Small Ranunculus, genome sequence, chromosomal, Lepidoptera

## Abstract

We present a genome assembly from an individual female *Hecatera dysodea* (the Small Ranunculus; Arthropoda; Insecta; Lepidoptera; Noctuidae). The genome sequence is 640.9 megabases in span. Most of the assembly is scaffolded into 32 chromosomal pseudomolecules, including the Z and W sex chromosomes. The mitochondrial genome has also been assembled and is 15.4 kilobases in length. Gene annotation of this assembly on Ensembl has identified 12,213 protein coding genes.

## Species taxonomy

Eukaryota; Metazoa; Ecdysozoa; Arthropoda; Hexapoda; Insecta; Pterygota; Neoptera; Endopterygota; Lepidoptera; Glossata; Ditrysia; Noctuoidea; Noctuidae; Hadeninae; *Hecatera*; *Hecatera dysodea* (Denis & Schiffermüller, 1775) (NCBI:txid988125).

## Background

*Hecatera dysodea*, known as the Small Ranunculus, is a moth with subtly attractive markings as an adult, and an interesting history of extinction and colonisation in the British Isles. Larvae feed on the seeds or flowers of lettuces, mainly Prickly Lettuce ( *Lactuca serriola*), but also other lettuce species, including cultivated varieties. Although *H. dysodea* has been reported as a pest of lettuces, as a seed and flower eater, they would only ever be eating bolted lettuces and thus a potential pest of lettuce seed crops. Adult moths visit flowers, especially of lettuces, and are readily attracted to light. Although it is sometimes reported as having one generation per year, Clancy *et al.*, 2012 report two overlapping generations, with adults on the wing from May to October.

Found naturally across mainly Central and Southern Europe and Central Asia, *H. dysodea* has also been accidentally introduced to the USA, where it is now widespread in the Pacific Northwest ( Landolt *et al.*, 2010). The species name ‘dysodea’ is thought to originate from the larvae, ‘ill-smelling’ ( Pratt, 1986), maybe a reference to the smell of the lettuce (they are not tasty after bolting). Chemical attractants are being used in the US to monitor and potentially control populations ( Landolt *et al.*, 2017).

The population of *H. dysodea* in England was always rather cyclical, with its heyday apparently around the end of the 19th century; thereafter there was a rapid decline with extinction in this country around the 1930s. Pratt (1986) summarised the history of the decline and loss of *H. dysodea* from Britain and suggested that more modern farming (i.e., fewer bolting lettuce plants), declines in market gardens in south-east England and a succession of wet summers could have combined to cause its local extinction. In 1997, moths were found again in Kent ( Agassiz & Spice, 1998) and *H. dysodea* has rapidly spread since then, now being rather widespread in England and in parts of Wales and its British population is classified as being of Least Concern ( Fox *et al.*, 2019). It is a species of rough, open ground, including brownfield sites and gardens.

## Genome sequence report

**Figure 1.**
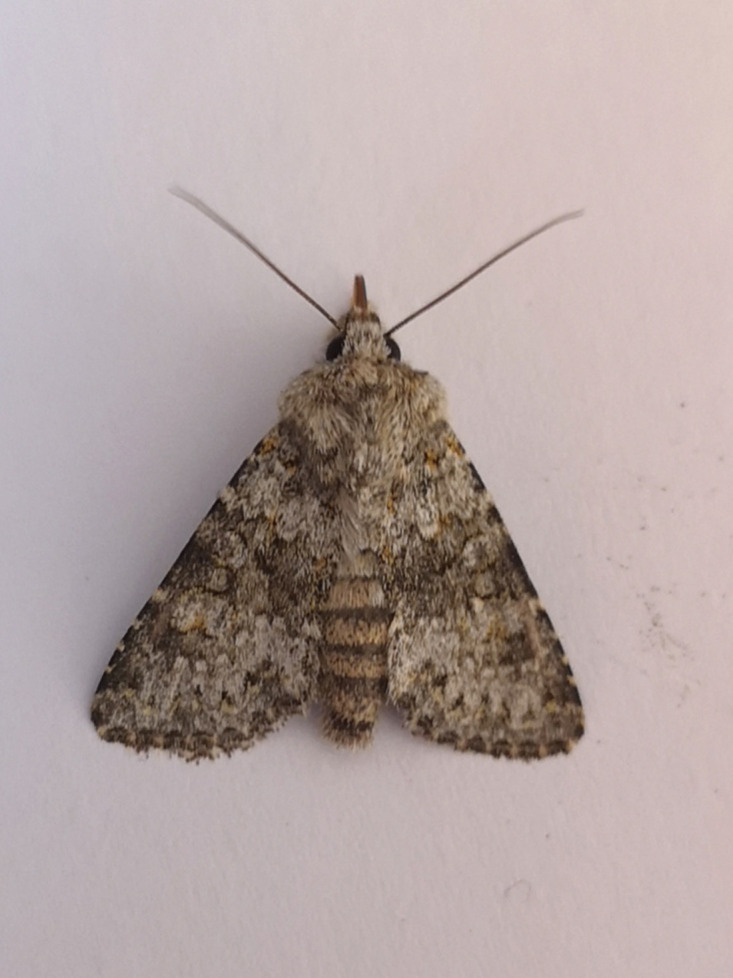
Photograph of the *Hecatera dysodea* (ilHecDyso1) specimen used for genome sequencing.

The genome was sequenced from one female *Hecatera dysodea* specimen ( Figure 1) collected from Tonbridge, UK (latitude 51.186305, longitude 0.286464). A total of 47-fold coverage in Pacific Biosciences single-molecule HiFi long reads and 60-fold coverage in 10X Genomics read clouds were generated. Primary assembly contigs were scaffolded with chromosome conformation Hi-C data. Manual assembly curation corrected 70 missing or mis-joins and removed six haplotypic duplications, reducing the assembly length by 0.55%% and the scaffold number by 47.37%, and increasing the scaffold N50 by 4.54%.

**Figure 2.**
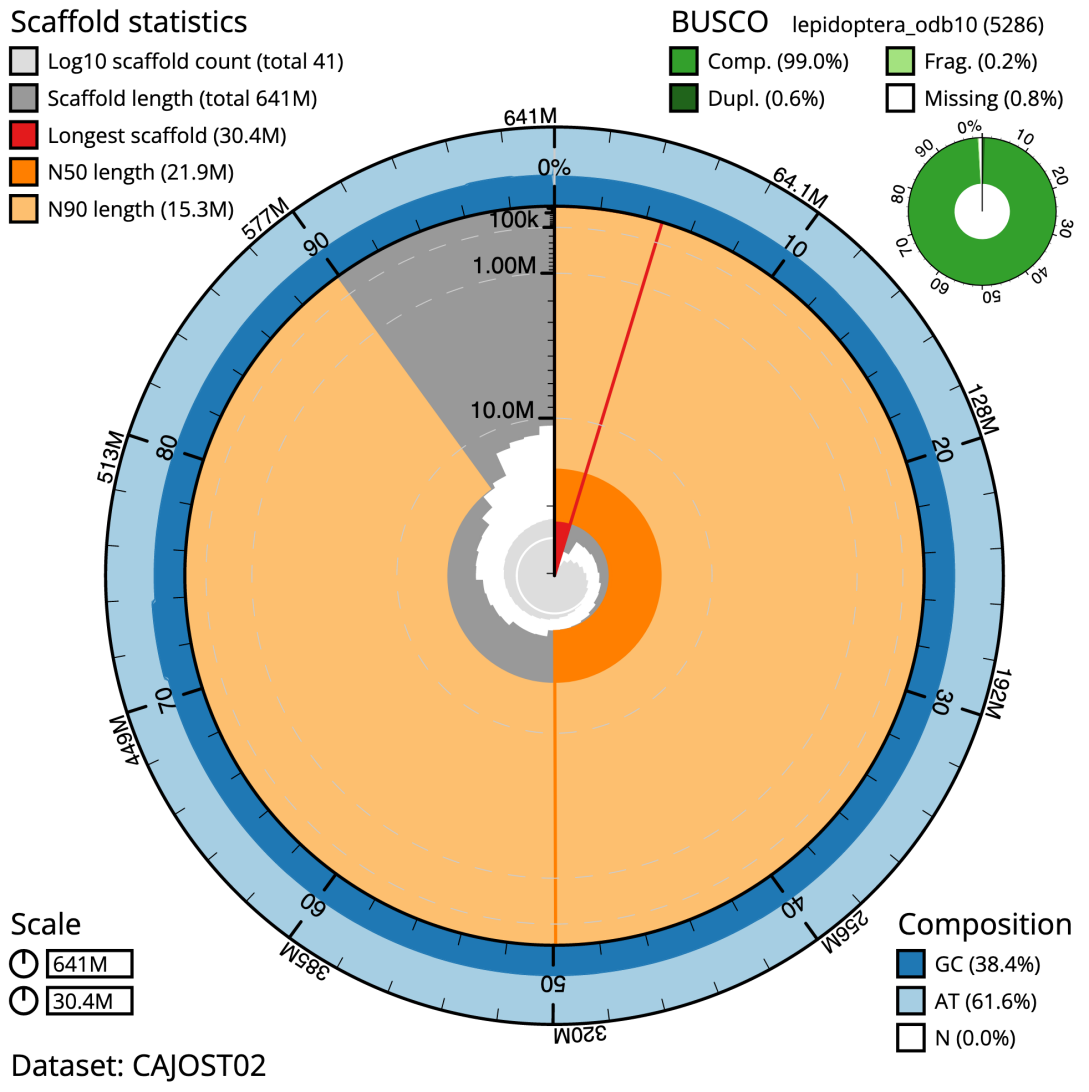
Genome assembly of *Hecatera dysodea*, ilHecDyso1.2: metrics. The BlobToolKit Snailplot shows N50 metrics and BUSCO gene completeness. The main plot is divided into 1,000 size-ordered bins around the circumference with each bin representing 0.1% of the 640,911,623 bp assembly. The distribution of scaffold lengths is shown in dark grey with the plot radius scaled to the longest scaffold present in the assembly (30,380,561 bp, shown in red). Orange and pale-orange arcs show the N50 and N90 scaffold lengths (21,896,367 and 15,320,587 bp), respectively. The pale grey spiral shows the cumulative scaffold count on a log scale with white scale lines showing successive orders of magnitude. The blue and pale-blue area around the outside of the plot shows the distribution of GC, AT and N percentages in the same bins as the inner plot. A summary of complete, fragmented, duplicated and missing BUSCO genes in the lepidoptera_odb10 set is shown in the top right. An interactive version of this figure is available at https://blobtoolkit.genomehubs.org/view/ilHecDyso1.2/dataset/CAJOST02/snail.

The final assembly has a total length of 640.9 Mb in 40 sequence scaffolds with a scaffold N50 of 21.9 Mb ( Table 1). Most (99.94%) of the assembly sequence was assigned to 32 chromosomal-level scaffolds, representing 30 autosomes and the W and Z sex chromosomes. Chromosome-scale scaffolds confirmed by the Hi-C data are named in order of size ( Figure 2– Figure 5; Table 2). The assembly has a BUSCO v5.3.2 ( Manni *et al.*, 2021) completeness of 99.0% (single 98.4%, duplicated 0.6%) using the lepidoptera_odb10 reference set.

**Table 1.** Genome data for *Hecatera dysodea*, ilHecDyso1.2.

Project accession data		
Assembly identifier	ilHecDyso1.2	
Species	*Hecatera dysodea*	
Specimen	ilHecDyso1	
NCBI taxonomy ID	988125	
BioProject	PRJEB43532	
BioSample ID	SAMEA7521514	
Isolate information	ilHecDyso1, female	
Assembly metrics *		*Benchmark*
Consensus quality (QV)	54	*≥ 50*
*k*-mer completeness	99.99%	*≥ 95%*
BUSCO **	C:99.0%[S:98.4%,D:0.6%], F:0.2%,M:0.8%,n:5,286	*C ≥ 95%*
Percentage of assembly mapped to chromosomes	99.94%	*≥ 95%*
Sex chromosomes	W and Z chromosomes	*localised homologous pairs*
Organelles	Mitochondrial genome assembled	*complete single alleles*
Raw data accessions		
PacificBiosciences SEQUEL II	ERR6406206	
10X Genomics Illumina	ERR6054504–ERR6054507	
Hi-C Illumina	ERR6054503	
PolyA RNA-Seq Illumina	ERR6464926	
Genome assembly		
Assembly accession	GCA_905332915.2	
Span (Mb)	640.9	
Number of contigs	115	
Contig N50 length (Mb)	18.0	
Number of scaffolds	40	
Scaffold N50 length (Mb)	21.9	
Longest scaffold (Mb)	30.4	
Genome annotation		
Number of protein-coding genes	12,213	
Number of non-coding genes	1,652	
Number of gene transcripts	21,454	

* Assembly metric benchmarks are adapted from column VGP-2020 of “Table 1: Proposed standards and metrics for defining genome assembly quality” from ( Rhie *et al.*, 2021). ** BUSCO scores based on the lepidoptera_odb10 BUSCO set using v5.3.2. C = complete [S = single copy, D = duplicated], F = fragmented, M = missing, n = number of orthologues in comparison. A full set of BUSCO scores is available at https://blobtoolkit.genomehubs.org/view/ilHecDyso1.2/dataset/CAJOST02/busco.

**Figure 3.**
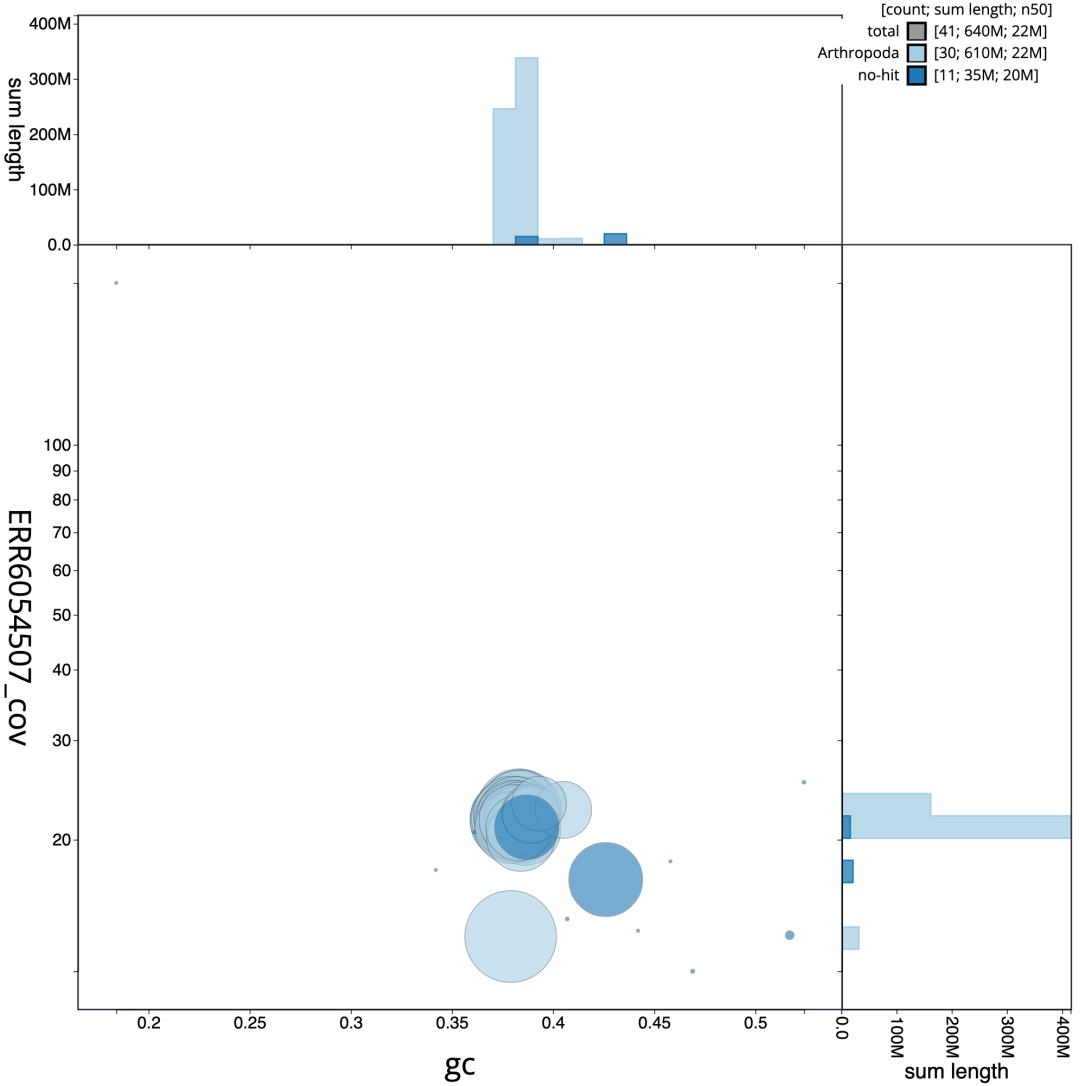
Genome assembly of *Hecatera dysodea*, ilHecDyso1.2: GC coverage. BlobToolKit GC-coverage plot. Scaffolds are coloured by phylum. Circles are sized in proportion to scaffold length. Histograms show the distribution of scaffold length sum along each axis. An interactive version of this figure is available at https://blobtoolkit.genomehubs.org/view/ilHecDyso1.2/dataset/CAJOST02/blob.

**Figure 4.**
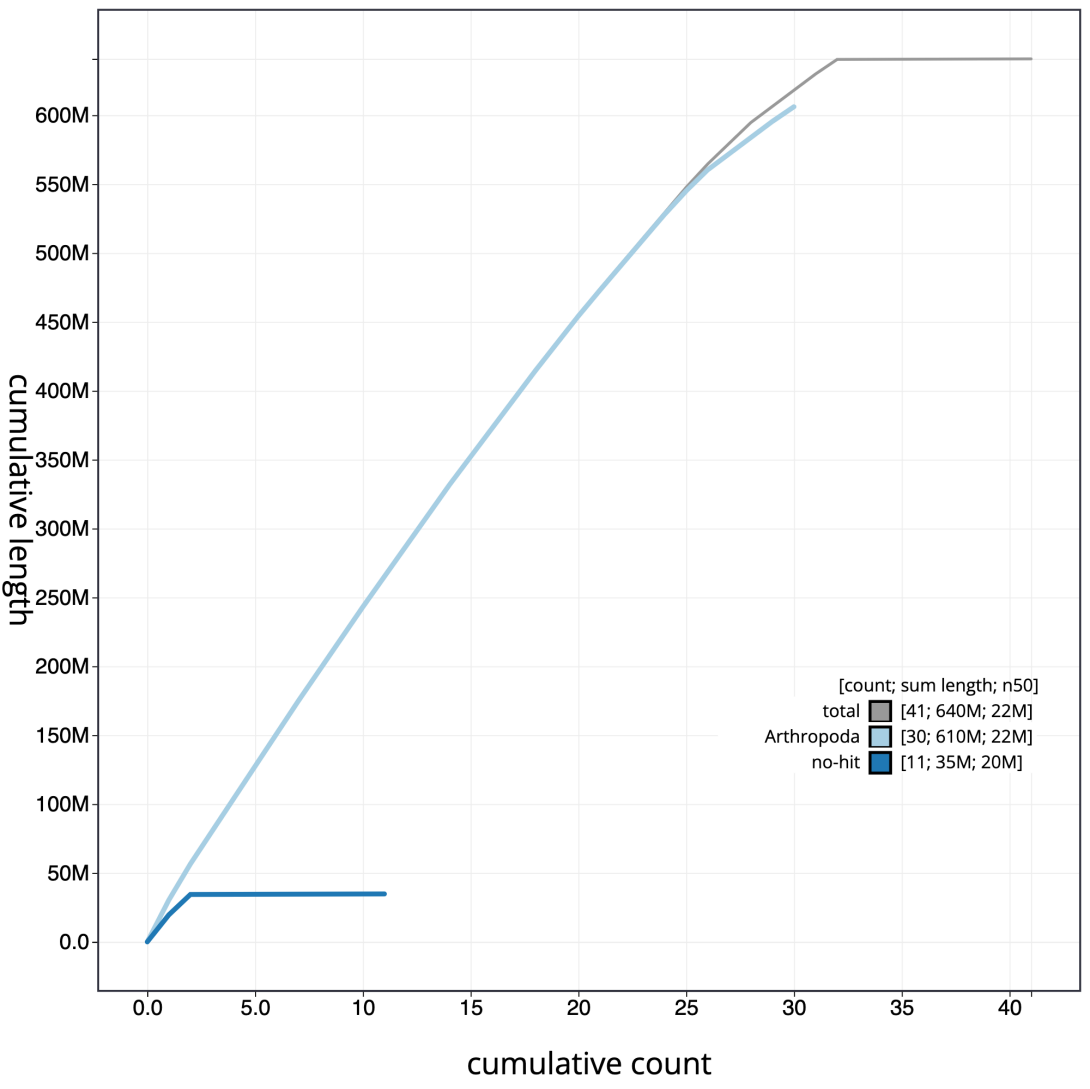
Genome assembly of *Hecatera dysodea*, ilHecDyso1.2: cumulative sequence. BlobToolKit cumulative sequence plot. The grey line shows cumulative length for all scaffolds. Coloured lines show cumulative lengths of scaffolds assigned to each phylum using the buscogenes taxrule. An interactive version of this figure is available at https://blobtoolkit.genomehubs.org/view/ilHecDyso1.2/dataset/CAJOST02/cumulative.

**Figure 5.**
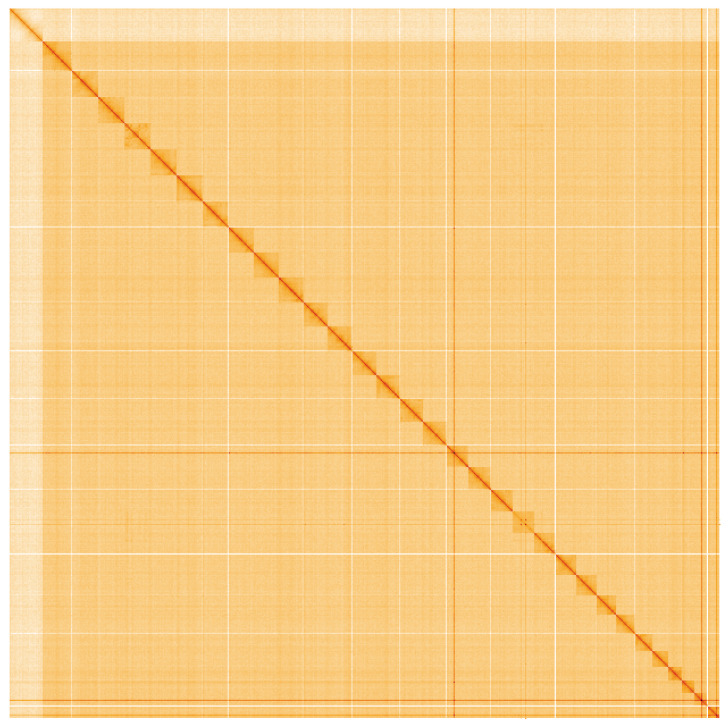
Genome assembly of *Hecatera dysodea*, ilHecDyso1.2: Hi-C contact map. Hi-C contact map of the ilHecDyso1.2 assembly, visualised using HiGlass. Chromosomes are shown in order of size from left to right and top to bottom. An interactive version of this figure may be viewed at https://genome-note-higlass.tol.sanger.ac.uk/l/?d=eT_0h2ElSVOAkIDCfeUuNA.

**Table 2.** Chromosomal pseudomolecules in the genome assembly of *Hecatera dysodea*, ilHecDyso1.

INSDC accession	Chromosome	Size (Mb)	GC%
HG995287.1	1	26.17	38.3
HG995288.1	2	23.96	38.3
HG995289.1	3	23.81	37.9
HG995290.1	4	23.58	38.3
HG995291.1	5	23.51	37.9
HG995292.1	6	23.29	38.4
HG995293.1	7	22.98	38.1
HG995294.1	8	22.89	38.1
HG995295.1	9	22.53	38.3
HG995296.1	10	22.25	38.3
HG995297.1	11	22.08	37.9
HG995298.1	12	22	38
HG995299.1	13	21.9	38.3
HG995300.1	14	21.03	38.2
HG995301.1	15	20.96	38
HG995302.1	16	20.95	38.1
HG995303.1	17	20.21	38.3
HG995304.1	18	20.07	38.2
HG995305.1	19	19.76	38.4
HG995307.1	20	18.7	38.6
HG995308.1	21	18.6	38.4
HG995309.1	22	18.3	37.9
HG995310.1	23	18.19	38.1
HG995311.1	24	16.97	38.4
HG995312.1	25	15.32	38.3
HG995313.1	26	14.75	38.7
HG995314.1	27	12.11	38.9
HG995315.1	28	11.61	38.9
HG995316.1	29	11.45	40.5
HG995317.1	30	10.61	39.3
HG995306.1	W	19.6	42.6
HG995286.1	Z	30.38	37.9
HG995318.2	MT	0.02	18.6
-	unplaced	0.39	48.1

## Genome annotation report

The *H. dysodea* genome assembly (GCA_905332915.2) was annotated using the Ensembl rapid annotation pipeline ( Table 1; https://rapid.ensembl.org/Hecatera_dysodea_GCA_905332915.2/). The resulting annotation includes 21,454 transcribed mRNAs from 12,213 protein-coding and 1,652 non-coding genes.

## Methods

### Sample acquisition and nucleic acid extraction

A female *Hecatera dysodea* (ilHecDyso1) was collected from Tonbridge, Kent (latitude 51.186305, longitude 0.286464) on 23 June 2020. The specimen was taken from a garden by Gavin Broad (Natural History Museum) using a light trap. The specimen was identified by Gavin Broad and preserved on dry ice.

DNA was extracted at the Tree of Life laboratory, Wellcome Sanger Institute (WSI). The ilHecDyso1 sample was weighed and dissected on dry ice with tissue set aside for Hi-C sequencing. Thorax tissue was disrupted using a Nippi Powermasher fitted with a BioMasher pestle. High molecular weight (HMW) DNA was extracted using the Qiagen MagAttract HMW DNA extraction kit. Low molecular weight DNA was removed from a 20 ng aliquot of extracted DNA using 0.8X AMpure XP purification kit prior to 10X Chromium sequencing; a minimum of 50 ng DNA was submitted for 10X sequencing. HMW DNA was sheared into an average fragment size of 12–20 kb in a Megaruptor 3 system with speed setting 30. Sheared DNA was purified by solid-phase reversible immobilisation using AMPure PB beads with a 1.8X ratio of beads to sample to remove the shorter fragments and concentrate the DNA sample. The concentration of the sheared and purified DNA was assessed using a Nanodrop spectrophotometer and Qubit Fluorometer and Qubit dsDNA High Sensitivity Assay kit. Fragment size distribution was evaluated by running the sample on the FemtoPulse system.

RNA was extracted from head tissue of (ilHecDyso1) in the Tree of Life Laboratory at the WSI using TRIzol, according to the manufacturer’s instructions. RNA was then eluted in 50 μl RNAse-free water and its concentration assessed using a Nanodrop spectrophotometer and Qubit Fluorometer using the Qubit RNA Broad-Range (BR) Assay kit. Analysis of the integrity of the RNA was done using Agilent RNA 6000 Pico Kit and Eukaryotic Total RNA assay.

### Sequencing

Pacific Biosciences HiFi circular consensus and 10X Genomics read cloud DNA sequencing libraries were constructed according to the manufacturers’ instructions. Poly(A) RNA-Seq libraries were constructed using the NEB Ultra II RNA Library Prep kit. DNA and RNA sequencing was performed by the Scientific Operations core at the WSI on Pacific Biosciences SEQUEL II (HiFi), Illumina HiSeq 4000 (RNA-Seq) and HiSeq X Ten (10X) instruments. Hi-C data were also generated from abdomen tissue of ilHecDyso1 using the Arima v2 kit and sequenced on the Illumina NovaSeq 6000 instrument.

### Genome assembly

Assembly was carried out with Hifiasm ( Cheng *et al.*, 2021) and haplotypic duplication was identified and removed with purge_dups ( Guan *et al.*, 2020). One round of polishing was performed by aligning 10X Genomics read data to the assembly with Long Ranger ALIGN, calling variants with freebayes ( Garrison & Marth, 2012). The assembly was then scaffolded with Hi-C data ( Rao *et al.*, 2014) using SALSA2 ( Ghurye *et al.*, 2019). The assembly was checked for contamination and corrected using the gEVAL system ( Chow *et al.*, 2016) as described previously ( Howe *et al.*, 2021). Manual curation ( Howe *et al.*, 2021). was performed using gEVAL, HiGlass ( Kerpedjiev *et al.*, 2018) and Pretext ( Harry, 2022). The mitochondrial genome was assembled using MitoHiFi ( Uliano-Silva *et al.*, 2022), which performed annotation using MitoFinder ( Allio *et al.*, 2020). The genome was analysed and BUSCO scores generated within the BlobToolKit environment ( Challis *et al.*, 2020). Table 3 contains a list of all software tool versions used, where appropriate.

**Table 3.** Software tools and versions used.

Software tool	Version	Source
BlobToolKit	3.5.2	Challis *et al.*, 2020
freebayes	1.3.1-17-gaa2ace8	Garrison & Marth, 2012
gEVAL	N/A	Chow *et al.*, 2016
Hifiasm	0.12	Cheng *et al.*, 2021
HiGlass	1.11.6	Kerpedjiev *et al.*, 2018
Long Ranger ALIGN	2.2.2	https://support.10xgenomics.com/genome-exome/software/pipelines/latest/advanced/other-pipelines
MitoHiFi	1	Uliano-Silva *et al.*, 2022
PretextView	0.2	Harry, 2022
purge_dups	1.2.3	Guan *et al.*, 2020
SALSA	2.2	Ghurye *et al.*, 2019

### Genome annotation

The Ensembl gene annotation system ( Aken *et al.*, 2016) was used to generate annotation for the *H. dysodea* assembly (GCA_905332915.2). Annotation was created primarily through alignment of transcriptomic data to the genome, with gap filling via protein to-genome alignments of a select set of proteins from UniProt ( UniProt Consortium, 2019).

### Ethics and compliance issues

The materials that have contributed to this genome note have been supplied by a Darwin Tree of Life Partner. The submission of materials by a Darwin Tree of Life Partner is subject to the Darwin Tree of Life Project Sampling Code of Practice. By agreeing with and signing up to the Sampling Code of Practice, the Darwin Tree of Life Partner agrees they will meet the legal and ethical requirements and standards set out within this document in respect of all samples acquired for, and supplied to, the Darwin Tree of Life Project. All efforts are undertaken to minimise the suffering of animals used for sequencing. Each transfer of samples is further undertaken according to a Research Collaboration Agreement or Material Transfer Agreement entered into by the Darwin Tree of Life Partner, Genome Research Limited (operating as the Wellcome Sanger Institute), and in some circumstances other Darwin Tree of Life collaborators.

## Data Availability

European Nucleotide Archive: Hecatera dysodea (small ranunculus). Accession number PRJEB43532; https://identifiers.org/ena.embl/PRJEB43532. ( Wellcome Sanger Institute, 2021) The genome sequence is released openly for reuse. The *Hecatera dysodea* genome sequencing initiative is part of the Darwin Tree of Life (DToL) project. All raw sequence data and the assembly have been deposited in INSDC databases. Raw data and assembly accession identifiers are reported in Table 1.

## Author information

Members of the Natural History Museum Genome Acquisition Lab are listed here: https://doi.org/10.5281/zenodo.4790043.

Members of the Darwin Tree of Life Barcoding collective are listed here: https://doi.org/10.5281/zenodo.4893703.

Members of the Wellcome Sanger Institute Tree of Life programme are listed here: https://doi.org/10.5281/zenodo.4783585.

Members of Wellcome Sanger Institute Scientific Operations: DNA Pipelines collective are listed here: https://doi.org/10.5281/zenodo.4790455.

Members of the Tree of Life Core Informatics collective are listed here: https://doi.org/10.5281/zenodo.5013541.

Members of the Darwin Tree of Life Consortium are listed here: https://doi.org/10.5281/zenodo.4783558.
