# Supplementary material for: The genome sequence of the Small Ranunculus, Hecatera dysodea (Denis & Schiffermüller, 1775)
Source: Wellcome Open Res. 2023 Feb 23;8:101. [Version 1] doi: 10.12688/wellcomeopenres.19067.1 (PMC10690034; doi:10.12688/wellcomeopenres.19067.1)
Supplement: Peer review history

# Reviewer report: Reviewer response for version 1

**DOI:** 10.21956/wellcomeopenres.21140.r70805

**Published:** 2024-06-20

**Authors:** Camille Cornet

**Affiliations:** 1 University of Neuchatel, Neuchâtel, Switzerland

The authors present a first chromosome-scale reference genome for the moth *Hecatera dysodea*, as part of the Darwin Tree of Life project. The assembly is of great quality in terms of completeness and contiguity. Notably, the sequenced individual was a female, therefore the W chromosome is assembled as well. This genome is likely to be a great resource for future use. I approve this genome note for indexing, after improvement of a few minor points:  

 1) The first paragraph of the background would benefit from a few references about the biology of the species (e.g. regarding the host plants and plants visited by the adults, especially since other *Noctuidae moths* are well known as nursery pollinators).

 2) “subtly attractive markings”: What does attractive mean in this instance?

 3) “It is a species of rough, open ground, including brownfield sites and gardens.” I suppose that this refers to the habitat of the species. This sentence would be more relevant in the first paragraph of the background rather than the very end, and would benefit from clarification (e.g., what is a rough ground?).

 4) The Hi-C yield (e.g. number of reads) should be mentioned in the first paragraph of the Genome sequence report.

 5) “… the assembly length by 0.55%%”: One of the percentage signs can be removed.

 6) In the methods: “…with tissue set aside for Hi-C sequencing”: Which tissue exactly was used for Hi-C?

 7) The software used to calculate the assembly metrics should be mentioned and cited (e.g. for consensus quality and k-mer completeness: Merqury I suppose?)

 8) In Table 1: Benchmarking for sex chromosomes is listed as “localised homologous pairs”. I supposed that this is a standard notation, but it is slightly confusing as the Z and W in Lepidoptera are not thought to be homologous.

 9) An important piece of information that is missing is how the sex chromosomes were identified. I supposed by mapping back the PacBio reads and comparing the coverage of chromosomes? Maybe for the Z by synteny analysis with a closely related species? This should be mentioned.

 10) In general, the Genome annotation methods are lacking some details. For example, there should be a mention of repeat masking before annotation, and it should be precise which UniProt protein set was used. In addition, the software used for annotation should be mentioned and cited.

Are sufficient details of methods and materials provided to allow replication by others?

Yes

Is the rationale for creating the dataset(s) clearly described?

Yes

Are the datasets clearly presented in a useable and accessible format?

Yes

Are the protocols appropriate and is the work technically sound?

Yes

Reviewer Expertise:

Evolutionary biology

I confirm that I have read this submission and believe that I have an appropriate level of expertise to confirm that it is of an acceptable scientific standard.

# Reviewer report: Reviewer response for version 1

**DOI:** 10.21956/wellcomeopenres.21140.r69731

**Published:** 2023-11-30

**Authors:** Zhijun Zhang, Yunsheng Wang

**Affiliations:** 1 Zhejiang Academy of Agricultural Sciences, Hangzhou, Zhejiang, China; 2 Bioinformatics, College of Plant Protection, Hunan Agricultural University, Changsha, Hunan, China

This is the firstly high quality genome assembly report for the moth species *Hecatera dysodea*. The authors have used appropriate sequencing and assembly strategies to generate a chromosome-level genome with excellent completeness and contiguity. I recommend this paper be accepted for publication after addressing the following minor comments:

 1.The sample acquisition and sequencing methods are clearly described. More details on the genome assembly process would be useful - for example, the coverage cutoff used for purging haplotigs with purge_dups.

 2.It would be useful to describe TE annotation process.

 3.The last sentence of the Background section is unclear and seems out of place. Consider revising or removing this sentence.

 The high quality genome sequences of Hecatera dysodea and will be a useful resource for further studies of this species' biology and demography. The data is openly available and clearly presented. I recommend accepting this paper pending minor revisions.

Are sufficient details of methods and materials provided to allow replication by others?

Yes

Is the rationale for creating the dataset(s) clearly described?

Yes

Are the datasets clearly presented in a useable and accessible format?

Yes

Are the protocols appropriate and is the work technically sound?

Yes

Reviewer Expertise:

thrips genome analysis; Tomato spotted wilt virus\thrips \plant interaction; integrated pest management.

We confirm that we have read this submission and believe that we have an appropriate level of expertise to confirm that it is of an acceptable scientific standard.

# Reviewer report: Reviewer response for version 1

**DOI:** 10.21956/wellcomeopenres.21140.r66344

**Published:** 2023-09-04

**Authors:** Craig Michell

**Affiliations:** 1 Red Sea Research Center, King Abdullah University of Science and Technology, Thuwal, Makkah Province, Saudi Arabia

This article is a genome assembly report for the species *Hecatera dysodea*, the small Ranunculus. It is a species of moth with an interesting demographic history in the British Isles and a recent introduction to North Western United States. The Authors use a combination of high coverage PacBio HiFi sequencing, 10X read cloud, Hi-C and RNA sequencing to assemble the genome and annotate the encoded genes.

 The sequencing strategy and protocols have produced a high quality genome with >99% of the assembly assigned to the correct number of chromosomes, with good contiguity and completeness. The quality metrics of the genome assembly are presented in Tables 1 and 2, as well as visually in Figures 2 through 5.

 The methods used to assemble the genome are briefly described; the software and versions of the programs are contained in Table 3.

 All of the data used to assemble the genome is available in public databases and the accessions can be found in Table 1 and the Data availability section.

 My comments are all only minor and they are found below.

 Comments:

1. The last sentence in the background is unclear. I do not know what this is referring to. “It is a species of rough, open ground, including brownfield sites and gardens.” It feels like an edit that was left in during different versions.
2. The methods are clearly described for the sample acquisition and extracts, but extremely brief for the genome assembly. It would be useful to know the settings and options used for each step, for example, purge_dups what coverage level was set for the cutoffs? The setting for this affects the removal of haplotigs.
3. Similarly, I would be interested to know the changes after polishing as it is generally not recommended to polish HiFi assemblies as it can reduce contiguity and break phase blocks. But maybe it is different with Long Ranger.

Are sufficient details of methods and materials provided to allow replication by others?

Yes

Is the rationale for creating the dataset(s) clearly described?

Yes

Are the datasets clearly presented in a useable and accessible format?

Yes

Are the protocols appropriate and is the work technically sound?

Yes

Reviewer Expertise:

Genome assembly of non-model organisms.

I confirm that I have read this submission and believe that I have an appropriate level of expertise to confirm that it is of an acceptable scientific standard.
